# Modeling and Experimental Studies on Polymer Melting and Flow in Injection Molding

**DOI:** 10.3390/polym14102106

**Published:** 2022-05-21

**Authors:** Krzysztof Wilczyński, Krzysztof J. Wilczyński, Kamila Buziak

**Affiliations:** 1 Polymer Processing Department, Faculty of Mechanical and Industrial Engineering, Warsaw University of Technology, Narbutta 85, 02-524 Warsaw, Poland; kamila.buziak@pw.edu.pl; 2 Politech Ltd., 86-031 Osielsko, Poland; wilczynski_k@wp.pl

**Keywords:** polymers, injection molding, modeling, melting

## Abstract

Injection molding, in addition to extrusion, is the most important technology in the polymer processing industry. When modeling injection molding, the global approach is necessary to take into account the solid polymer transport, polymer melting and the polymer melt flow. The model of polymer melting is fundamental for the development of such a global injection molding model. In the paper, the state-of-the-art of modeling and experimentation of the flow and melting in injection molding machines has been presented and discussed. It has been concluded that the existing mathematical models have no strong experimental basis. Therefore, experimentation of the polymer flow and melting in the injection molding machine has been performed, and the effect of processing conditions: the screw speed, the plasticating stroke and the back pressure on the process course has been investigated. Starving in the beginning sections of the screw has been observed, which was not presented in the literature so far. The novel concepts of injection molding modeling have been discussed.

## 1. Introduction

At present, the polymer processing designing is aided by computer process simulations. Modeling allows you to predict the course of the process on the basis of processing conditions, i.e., material characteristics, process operating parameters and machine geometry data.

An immense number of different types of polymeric materials and the variety of their applications require the use of various processing methods, among which extrusion and injection molding play a special role. Extrusion is the most mass technology in the polymer processing industry since more than half of all plastics are extruded. Extrusion is also fundamental for compounding, i.e., mixing, pelletizing, filling, reinforcing, etc. Injection molding is suited well for mass production of parts of complex shapes and precise dimensions. About one-third of all thermoplastics are injection molded.

Extrusion is the process of continuous forming of polymer products, the so-called profiles, e.g., pipes, rods, plates, films, etc. Extrusion consists of the continuous melting of a polymer in the plasticating unit of the extruder and then pushing (extruding) this melted material, under the pressure developed in the plasticating unit, through the openings of the forming tool that is called the die. Melting occurs as a result of the polymer heating up by the extruder heating system and as a result of energy dissipation in the extruded material.

Injection molding is a cyclic process of forming polymer products called moldings. It consists of cyclic melting of a polymer in the plasticating unit of the injection molding machine and then injecting the melted polymer, under the pressure developed in the plasticating unit, into the injection mold, shaping the product. Similar to extrusion, melting takes place here as a result of the polymer heating up by the heating system and as a result of energy dissipation in the material.

**Figure 1:**
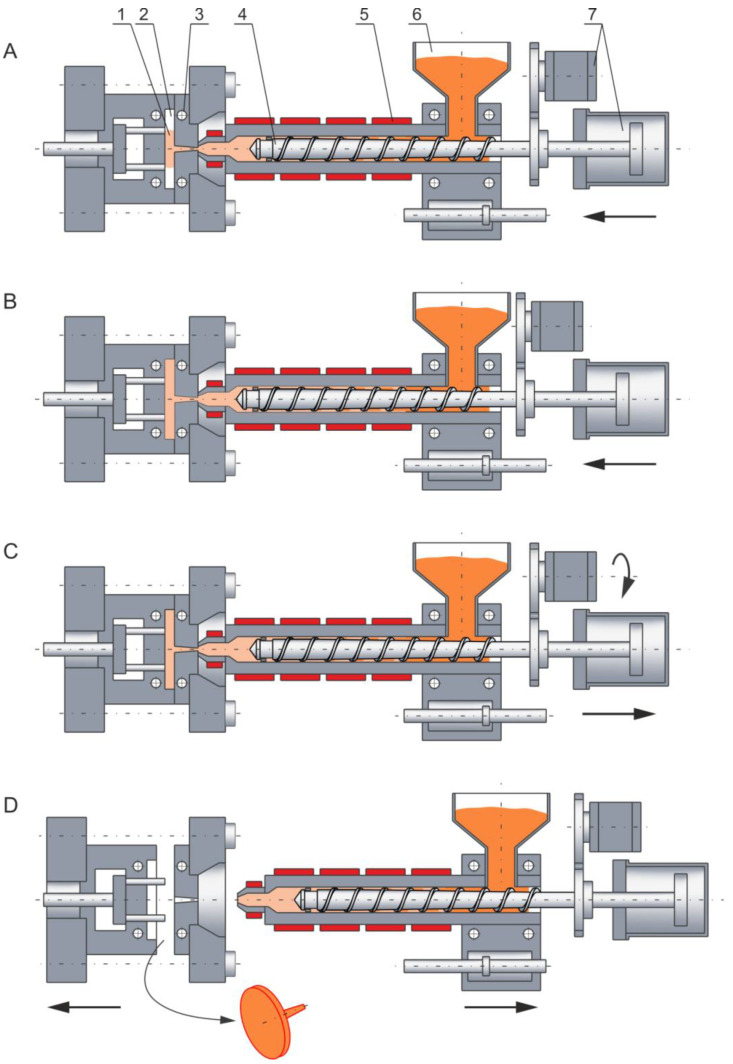
Injection molding process: (**A**)—injection (mold filling), (**B**)—holding, (**C**)—plasticating (melting), (**D**)—mold opening and molding (part) ejection; 1—molding (part), 2—mold cavity, 3—cooling channels, 4—screw, 5—heaters, 6—hopper, 7—screw drive system (with permission from *Rheology in Polymer Processing. Modeling and Simulation* by K. Wilczyński; Carl Hanser Verlag: Munich 2021 [1]).

The course of the injection molding process is depicted in Figure 1. The plasticating unit is the basic component of the injection molding machine, and it performs functions similar to those in the extruder. However, there are essential differences here. The rotating screw conveys the molten polymer to the screw front, and then the polymer is injected into the mold through the axial displacement of the screw.

When modeling extrusion and injection molding, the global approach is necessary to take into account the solid polymer transport, polymer melting and the polymer melt flow. The model of polymer melting is fundamental for the development of such global models.

When modeling the polymer melt flow in injection molds, the input data are basically unknown, for example, the polymer temperature. Thus, a global model of the injection molding process is needed that would describe both the flow in the injection molding machine and in the mold. The process output parameters from the plasticating unit would be the input parameters for the mold.

The first modeling approaches for screw processing of polymers were made for extrusion. The first analysis of flow in single screw machines were performed by Rowell and Finlayson [2] and later by Carley et al. [3,4], who described the drag flow and pressure flow for an isothermal Newtonian fluid. Solid conveying was modeled first by Darnell and Mol [5], who described material transport and pressure development.

**Figure 2:**
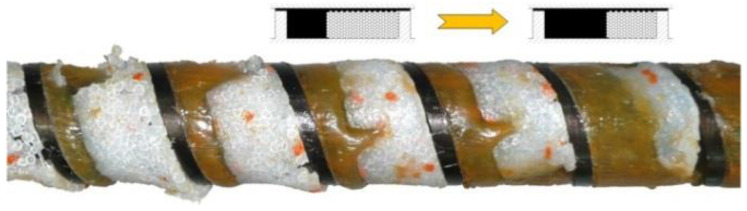
Melting mechanism (CSM) at flood fed single screw extrusion of polypropylene [8].

The first melting studies in the single screw extruder were carried out by Maddock and Street [6,7], who used the technique of pulling out the screw from an extruder. They analyzed the cross-sections of the polymer removed from the screw and observed the melting mechanism. According to this mechanism, the so-called contiguous solid melting (CSM), a melt layer was formed between the barrel and the solid material, which was scrapped off by the transverse flow in the screw and accumulated at the active screw flight. The solid was gradually diminished by the effects of heat conduction from the barrel and viscous dissipation in the polymer melt (Figure 2). Thus, two steps of melting were distinguished, the so-called pre-melting zone (or delay zone), corresponding to the formation of the polymer melt layer, and the melting zone (or plasticating zone), corresponding to the accumulation of the polymer melt at the active screw flight.

Tadmor et al. [9,10,11] did a similar experiment and proposed the first model of polymer melting in extrusion, which allowed for the elaboration of the first computer model of polymer extrusion EXTRUD [12]. This polymer melting model was based on the computation of the velocity and temperature distribution in the polymer melt film and temperature distribution in the polymer solid bed (Figure 2). Then, a mass balance in the polymer melt film and the polymer solid, as well as an energy balance at the interface melt film/solid bed, were carried out. Finally, the melting rate was predicted. Later, more detailed models were proposed for solid conveying [13,14] and for the pre-melting zone (or delay zone) [15]. Thus, the extrusion model has been substantially improved. Studies of polymer melting performed by other researchers confirmed the Tadmor model.

Many books have been written on extrusion, for example, by Fischer [16], Mc Kelvey [17], Schenkel [18], Fenner [19], Tadmor and Klein [20], Hensen et al. [21], White [22], White and Potente [23], Tadmor and Gogos [24], White and Kim [25], Rauwendaal [26], Lafleur and Vergnes [27], Agassant et al. [28], Chung [29], Campbell and Spalding [30] and Vlachopoulos and Polychronopoulos [31].

The issue of modeling the extrusion has been presented in books, for example, by White and Potente [23], Rauwendaal [26], Agassant et al. [28], and has been reviewed in papers by Arrifin et al. [32], Wilczyński et al. [33], Teixeira et al. [34], Malik et al. [35], Wilczyński et al. [36], Hyvärinen et al. [37], Lewandowski et al. [38] and Marschik et al. [39].

**Figure 3:**
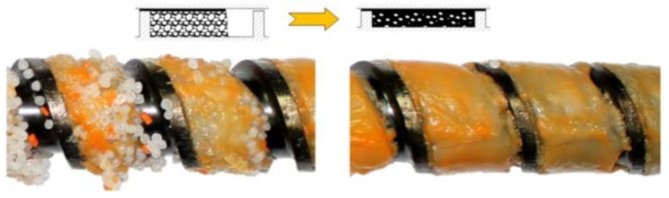
Melting mechanism at starve fed single screw extrusion of polypropylene [8].

The flood fed single screw extrusion was widely studied and modeled; however, little information was available on the starve fed extrusion. The first studies were carried out by Lopez-Latorre and McKelvey [40] and later by Isherwood et al. [41], Strand et al. [42] and Thompson et al. [43]. Recently, Wilczyński et al. [8,44] performed extensive experimental studies on the starve fed extrusion and proposed the mechanism and model of polymer melting. Then, they built the first computer model of this process, SSEM-Starve [45]. Two stages of melting have been distinguished. In the partially filled section of the screw, the polymer granules were collected at the active screw flight and were melted by conduction, mainly. In the fully filled section, the unmolten solid particles were suspended in the earlier molten polymer, and melting progressed by heat dissipation (Figure 3). This model was later extended to the non-conventional screw configurations [46,47] and to the extrusion of polyblends and composites [48,49,50].

**Figure 4:**
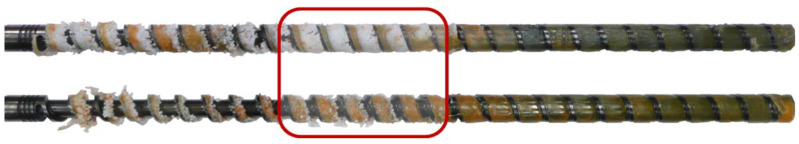
Polymer flow in the flood fed/starve fed single screw extrusion [8].

The polymer flow in the classical flood fed extrusion in comparison with the polymer flow in the starve fed extrusion is depicted in Figure 4. It is worth noting that the difference in the flow rate (extrusion throughput) was very small here, about 2%. However, the filling of the screw was significantly different. A detailed discussion of this issue has been presented in [51] based on the extensive process simulations and experimentations.

The research on melting in twin-screw extruders was much more limited. It is worth noting here that twin screw extrusion is performed with metered feeding, mainly, thus with clear starving.

The studies were performed mainly for co-rotating twin-screw extruders, both by experimentation, by Bawiskar and White [52], Todd [53], Sakai [54] and Gogos [55,56,57], and by modeling. Potente and Melish [58], as well as Bawiskar and White [59], developed the models based on the classical Tadmor model [20] while assuming the gradual forming of the molten layer from the barrel toward the screw. Bawiskar and White [59] described the formation of two stratified layers of polymer melt in contact with the barrel and solid pellets in contact with the colder screw. Potente and Melisch [58] considered the melting of particles uniformly suspended in the polymer melt. Vergnes et al. [60,61], as well as Zhu et al. [62], developed dispersive models based on the analysis of the flow of the solid/melt mixture of an equivalent viscosity. Based on these investigations, the global models of the co-rotating twin screw extrusion were built [34,63,64,65,66,67,68]. This issue was reviewed and discussed by Teixeira [69].

**Figure 5:**
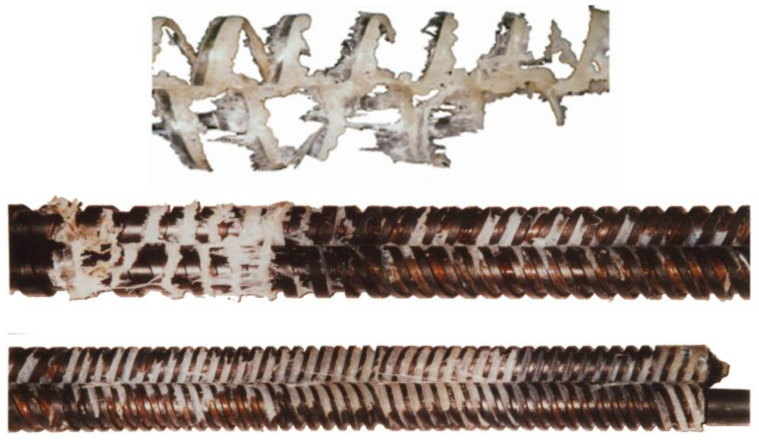
Melting mechanism observed in counter-rotating twin screw extrusion [74].

Melting in counter-rotating twin-screw extruders is much less known. The first observations were presented by Janssen [70]. White et al. [71,72] noticed that melting occurs here more rapidly than in co-rotating extruders. Wilczyński and White [73] revealed the mechanism of melting in counter-rotating twin-screw extruders. They observed that melting was initiated both between the screws and at the barrel. The melting between the screws was induced by frictional work on the granules by the calendering stresses between the screws. The melting at the barrel was initiated by the barrel temperature higher than the melting point and was propagated by the viscous dissipation heating of the melt film. These observations (Figure 5) allowed us to develop models of melting in both of those regions [74]. Further studies of melting were reported by Wang and Min [75,76] as well as by Wilczyński et al. [77]. Based on these studies, the global models of the counter-rotating twin screw extrusion were built [78,79,80,81].

The methods of investigating and modeling the polymer flow and melting in the extrusion process were adopted to modeling of these in injection molding. However, while many books and review papers have been devoted to the modeling of extrusion, there has been much less to injection molding. These were mainly limited to modeling the flow in injection molds without considering the polymer flow and melting in the plasticating unit, e.g., by Manzione et al. [82], Kennedy and Zheng [83], Kamal et al. [84], Osswald et al. [85] and Wang et al. [86]. Other books have been devoted to the mold design, e.g., by Menges et al. [87], Rees [88], Unger [89], Mennig and Stoeckhert [90], Kazmer [91], Turng and Chen [92], Beaumont [93] and Catoen and Rees [94], or to the injection molding machines by Johannaber [95]. An important review paper on modeling and optimization of injection molding was presented by Fernandes et al. [96]. At present, the important software for simulating the flow in injection molds are MOLDFLOW [97], Moldex3D [98] and CADMOULD [99], by example recently used in [100,101]. Simulations of the polymer flow in injection molds were also performed using the CFD software COMSOL Multiphysics [102] with an example of [103].

The first experimental studies of melting in injection molding machines were carried out by Donovan et al. [104] using the “screw pulling out technique”. They revealed that the screw recharge process was the transient plasticating extrusion which gradually approached the equilibrium extrusion behavior as the screw rotated. If the time of screw rotation was a high fraction of the total cycle time, the plasticating behavior was similar to the extrusion behavior, but if the time of screw rotation was a small fraction of the total cycle time, the plasticating behavior was substantially different.

The reports were also presented on the experimental study of the solid bed transport and melting in the screw channel of the injection molding machines [105,106,107] with the use of “transparent windows” made in the barrel to observe the polymer flow.

Important experimental studies on polymer melting in injection molding machines were carried out by Gao et al. [105]. They developed the visual system to observe the polymer flow in the reciprocating screw injection molding machine. The effects of processing conditions on the polymer melting were studied, and it was concluded that the modeling of the plasticating phase cannot be treated simply as an extrusion process.

Different barrel temperatures, plastication strokes, screw rotation speeds, and back pressures were used to observe their effects on the melting behavior.

It was observed that there was more polymer melted at a lower screw speed than at a higher speed. As the plastication stroke was the same, a lower speed resulted in longer plastication time, while a higher speed resulted in shorter plastication time.

It was also seen that for the case of the lower plastication stroke, the melting was faster, while for the case of the higher plastication stroke, the polymer melting length was longer. With a small injection stroke, the injection volume was small in comparison to the amount of material enclosed in the screw channel. As a result, the residence time was comparatively long, and hence melting was primarily due to heat conduction. Conversely, with a long injection stroke, the injection volume was large in comparison with the screw channel volume, and the melting was primarily due to the shearing.

It is known that the back pressure affects the melting, temperature distribution, and pressure distribution in the screw channel. Different back pressures result in different pressure profiles in the screw channel and different plastication times. It was observed that an increase in back pressure was advantageous to the melting. A higher back pressure also led to a larger left-hand melt pool size than a lower back pressure.

An increase in the dwelling time results in more heat being conducted and consequently melts more polymer.

A comparison between the barrel temperature and the temperature at the interface of the polymer and barrel showed that the polymer-barrel interface temperature was higher than the barrel temperature, even more than 20 °C. This resulted from the combination effects of shear heating at the polymer-barrel interface and the barrel heat loss to the environment. It is worth noting that in injection molding modeling, the polymer temperature at the wall is commonly assumed to be the same as the barrel temperature, which is not an accurate reflection of reality.

Modeling of plasticization during injection molding has been a complex problem, particularly for the solid conveying section. This can be further complicated by a break-up of the solid bed due to the reciprocation motion in the process. The break-up of the solid bed can introduce inhomogeneities in the moldings. The solid bed break-up in a reciprocating screw was experimentally studied using a transparent barrel system designed by Jin et al. [106]. It was observed that the break-up occurred in the latter melting stage, where the solid bed strength was reduced because of the continuous reducing the solid bed thickness, where also the melt pressure was high. The onset of break-up was dependent on the process conditions. It was observed that the solid bed broke easier at the higher screw rotation speed and that the longer injection stroke, the higher backpressure and the longer dwell time. The break-up typically started by forming a slit or slits that were nearly perpendicular to the screw flight. It is worth noting that the properly designed barrier screw may be effectively used to prevent solid-bed break-up.

Other visual studies were performed by Pham et al. [107]. The results confirmed the usual view of a contiguous solid bed melting process. It was observed that the solid bed width increased with the screw rotation speed, as predicted by a classical theory. The solid bed break-up was seen at every screw speed. Conductive melting from the flight side of the solid bed was also viewed. The solid bed velocity was distinct from the screw velocity.

Donovan [108,109] was the first who proposed the model for polymer melting in the injection molding process. This was a heuristic model which required experimental evaluation of an empirical parameter specific to a particular polymer over the studied range of operating conditions. This model was based on the steady-state extrusion model [110].

Lipshitz et al. [111] proposed the model for melting, which was based on the detailed physical mechanisms taking place in the reciprocating screw injection molding machine. This model permitted the computation of the solid bed profile as a function of time during the injection cycle. It consisted of the dynamic extrusion melting model for the rotation period, the transient heat conduction model with a phase transition for the screw rest period, and the model for the drifting of the beginning of melting during the injection cycle.

Several papers have been published on the modeling of plasticating in injection molding machines. Rauwendaal [112,113] studied the effects of axial screw movement on solids and melt conveying in reciprocating extruders. Dormeier and Panreck [114] briefly discussed the melting process when the screw stopped in the reciprocating extruder. Rao [115] developed a relatively simple model for describing the plasticating process in a reciprocating injection molding screw by combining the melting model for extrusion of Tadmor (20) with the conduction melting model of Donovan (108).

Potente [116] presented the mathematical approach to simulate the polymer melting in injection molding. However, this model did not take into account the solid conveying and transient sections and used some special modeling empirical constants. Later, he calculated the power requirement of the plasticating systems of the injection molding machines and the extruders [117].

Bereaux et al. [118] developed a simple model of throughput and pressure development for single screw extruders and injection molding machines. The model is based on viewing the entire screw simply as a pump, conveying a solid fraction and a molten fraction. This concept results from that during most of the melting process, a conveying of the solid material side by side with the melt takes place. This two-phase conveying can be drawn to the one-phase flow model of a melt pump by assuming that the pressure gradient in the screw channel is solely due to melt flow, but the flow rate at any cross-section can be considered as the sum of the solid bed flow rate and the melt pool flow rate.

At that time, no studies existed for modeling the transient melting process in the feeding stage (with rotating screw), the injection stage and the whole cycle of injection molding.

Yung et al. [119,120,121] were the first who considered the whole cycle of the process, which comprised of three stages: feeding (screw rotating and moving backward), stopping (no screw movement) and injecting (screw moving forward without rotation) and developed the transient models for the melting process in these.

Another comprehensive model of plasticization in injection molding, which reflects well the process dynamics, was developed by Steller and Iwko [122,123]. This model takes into account the periodical action of the screw, to-and-from screw motion with controlled stroke and static and dynamic melting. However, this model does not take into account the existence of a delay zone, uses a simplified melting model, and the equations of energy and momentum are solved analytically. More details of this model were published in [124,125,126].

A few years ago, a novel model for the plasticization process of injection molding was proposed by Fernandes et al. [127]. In this study, the software developed by Gaspar-Cunha [128] for modeling the extrusion process was modified to take into account the backward movement of the screw, the presence of a non-return valve and the heat conduction during the idle time. The model used the Tadmor approach [20] for temperature computation during the plasticating and 3D transient equation of heat conduction for the polymer in the injection chamber during the idle times. The computation of the pressure profile was done by force and torque balances on a differential screw channel element [20]. The forces included friction between the barrel and the solid bed, friction due to the contact of the solids with the screw root and screw walls and normal reactions and forces due to the pressure gradient. The model was used to study the effect of some important process parameters, such as barrel temperature profile, screw speed, backpressure and flow rate during injection and cycle times. An experimental assessment of the computational results was also done.

Recently, a comprehensive model of injection molding has been presented by Iwko et al. [129]. The main assumptions of this model are the following:

- The process is quasi-steady;
- The polymer melting contains two phases: the static melting at the stationary screw and the dynamic melting at the rotating screw with its axial backward movement;
- The screw retreat is computed by the equality of the calculated pressure in the front of the screw and the back pressure;
- Three zones of the plasticating system are distinguished: the feed port and the solid conveying zone, the delay zone, as well as the melting and melt conveying zone.

The starting point for modeling was the model of steady state extrusion, which is similar to the classical extrusion model of Tadmor [20]. However, in contrast to the steady conditions characteristic of extrusion, the lengths and positions of dynamical zones change in time within the injection cycle. So as to describe these changes, it was assumed that two coupled states are valid during the cycle: at the end of screw rotation, i.e., at the beginning of static melting, and at the beginning of screw rotation, i.e., at the beginning of dynamic melting.

The model makes it possible to predict the most important process characteristics, such as the solid bed profile, the pressure and temperature distributions, the mass flow rate, the power consumption and the screw torque. The model was verified experimentally for five thermoplastic polymers during the injection molding with different back pressures, screw speeds, dwell times and barrel temperatures. The important output process parameters, such as the temperature and pressure profiles, the power demand by the screw, the torque on the screw and the screw rotation time, were measured. It was found that the model correctly determines the dynamics of the plasticization process under the changes in the most important input parameters.

It follows from the above literature data that the development of an adequate and comprehensive model of the melting process in injection molding has not been fully completed so far. It also results from the literature [36] that there exist several commercial software programs for the extrusion process, such as EXTRUD [20], SPR [130], NEXTRUCAD [131], REX [132,133], PASS [134], SSEM [135,136,137], or the research computer models developed by Fukase et al. [138], Zavadsky and Karnis [139], Vincelette et al. [140] and Amellal and Lafleur [141]. However, there is probably one computer program—PSI—available for the analysis of the injection molding process [142].

In summarizing, there are several models of injection molding (plasticating unit) describing the process with different levels of complexity, accuracy and scope of the description of process phenomena. These models were developed mainly based on the experiments and observations performed for the extrusion process and were verified experimentally by selected process parameters measurements, such as pressure and temperature profile, power consumption, etc. However, these were not related to the melting rate or solid bed profile.

It is worth noting that most of the research on the polymer flow in extrusion and injection molding is related to polymeric materials in the form of granules. Studies on the polymer melting and flow behavior of polymer powders instead of granules are very limited [143,144].

An early model of Donovan [108] was based on the injection molding studies with the “screw pulling-out technique”; however, the photographs of the screws removed from the barrel were not presented. Thus, there is an obvious lack of experimental studies on the polymer flow and melting in injection molding machines showing the filling of the screw and transport mechanism, especially in the beginning sections of the screw. The reason is that it is difficult to perform such an experiment, and it is especially difficult to quickly remove the screw from the injection molding machine in order to prevent the material from melting and to maintain the structure (state) of the material corresponding to the moment of stopping the machine.

Therefore, in this review paper, the experiments have been performed to evaluate the existing concepts of process modeling and to clear the physical basis of modeling. In the experiment, the “screw pulling-out technique” was applied to observe the polymer transport and melting. Based on these experiments, novel concepts of modeling have been proposed.

In general, the existing models of the injection molding process (plasticating unit) discussed above differ from the extrusion models in that they involve the static and dynamic phases of melting (stationary and rotating screw) with an axial screw movement. However, it is assumed that the screw is fully filled with a material as in the flood fed extrusion (Figure 2), which is inconsistent with our observation [36] where starvation is clearly seen as in the starve fed extrusion (Figure 3).

## 2. Experiment

### 2.1. Material and Process Data

The polystyrene Polystyrol 454H (manufactured by BASF, Ludwigshafen, Germany) was used in the research. The basic material data were as follows: the specific density ρ = 1.05 g/cm^3^, the melt flow index MFI = 4.0 g/10 min (M = 5.0 kg, T = 200 °C), and the Vicat temperature T_v_ = 85 °C (F = 50 N).

The injection molding machine FORMOplast 235/80 (manufactured by PONAR, Żywiec, Poland) was applied with the clamping force F_clamp_ = 80 T.

A conventional screw of the diameter D = 45 mm, the length L = 540 mm and the screw pitch t = 36 mm was used. The lengths of the screw sections were equal to L_F_ = 305 mm (the feeding sectrion), L_C_ = 135 mm (the compression section), L_M_ = 100 mm (the metering section). The depths of the screw channel in the sections were equal to H_F_ = 10 mm (the feeding section), H_M_ = 3.3 mm (the metering section) and the compression ratio, which is the ratio of the screw channel depth in the feeding section to the screw channel depth in the metering section was equal to CR = H_F_/H_M_ = 3.

Different processing conditions have been applied to study their effect on the polymer flow as well as melting in the injection molding machine. The screw rotation speeds were equal to N = 100 rpm and 300 rpm, the plastication strokes: h_plast_ = 1 D and 3 D, and the back pressures (hydraulic): P_back_ = 0 MPa, 2 MPa and 3 MPa.

The temperature was set at T_I_ = 190 °C, T_II_ = 200 °C, T_III_ = 220 °C in the barrel sections, and T_nozzle_ = 230 °C in the nozzle.

### 2.2. Results

The results of the experiments are depicted in Figure 6, Figure 7, Figure 8, Figure 9, Figure 10, Figure 11, Figure 12, Figure 13, Figure 14, Figure 15, Figure 16, Figure 17, Figure 18 and Figure 19. An important observation is that in all the cases under study, the screw channel was not fully filled in the beginning section of the screw, and starvation was clearly seen. A yellow mark means the region of starving.

**Figure 6:**
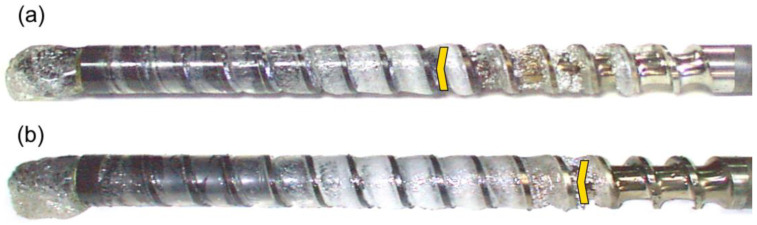
An effect of the screw speed on the polymer flow in the injection molding process at the plastication stroke h_plast_ = 1 D, and the back pressure P_back_ = 0 MPa: (**a**) N = 100 rpm, (**b**) N = 300 rpm.

**Figure 7:**
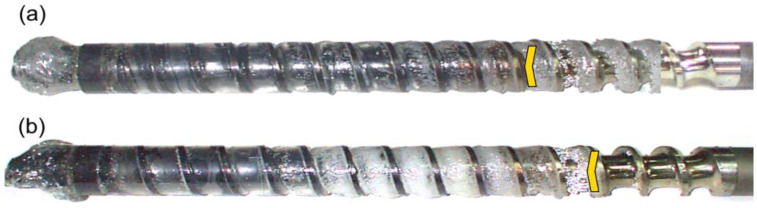
An effect of the screw speed on the polymer flow in the injection molding process at the plastication stroke h_plast_ = 1 D, and the back pressure P_back_ = 2 MPa: (**a**) N = 100 rpm, (**b**) N = 300 rpm.

**Figure 8:**
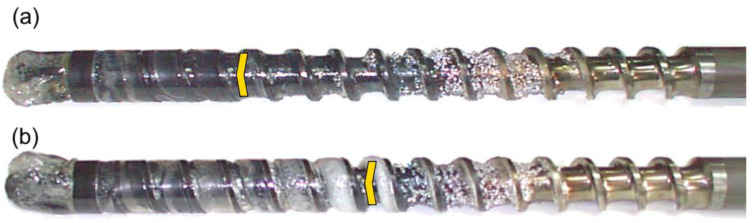
An effect of the screw speed on the polymer flow in the injection molding process at the plastication stroke h_plast_ = 3 D, and the back pressure P_back_ = 0 MPa: (**a**) N = 100 rpm, (**b**) N = 300 rpm.

**Figure 9:**
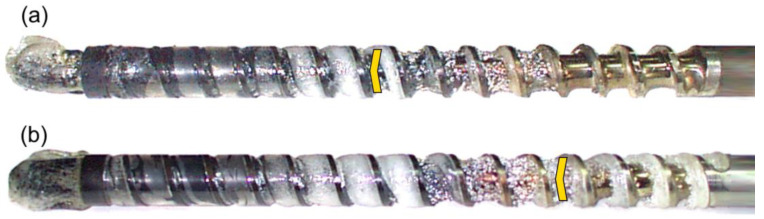
An effect of the screw speed on the polymer flow in the injection molding process at the plastication stroke h_plast_ = 3 D, and the back pressure P_back_ = 2 MPa: (**a**) N = 100 rpm, (**b**) N = 300 rpm.

**Figure 10:**
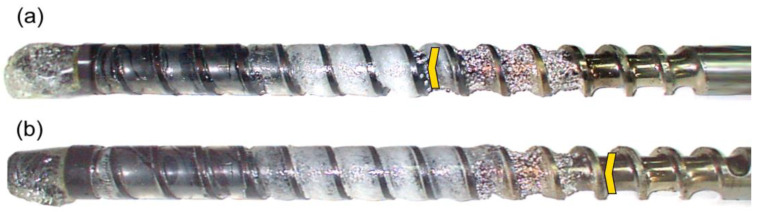
An effect of the screw speed on the polymer flow in the injection molding process at the plastication stroke h_plast_ = 3 D, and the back pressure P_back_ = 3 MPa: (**a**) N = 100 rpm, (**b**) N = 300 rpm.

An effect of the screw speed (N = 100 rpm and 300 rpm) on the polymer flow in the injection molding process at the constant plastication stroke and the constant back pressure is shown in Figure 6, Figure 7, Figure 8, Figure 9 and Figure 10. It is seen that when screw speed increases, the melting slows, which results from the shorter plastication time. It is also seen that the starvation decreases since more polymer is delivered in front of the screw tip.

**Figure 11:**
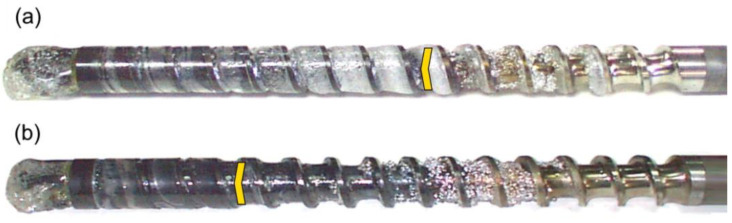
An effect of the plastication stroke on the polymer flow in the injection molding process at the screw speed N = 100 rpm, and the back pressure P_back_ = 0 MPa: (**a**) h_plast_ = 1 D, (**b**) h_plast_ = 3 D.

**Figure 12:**
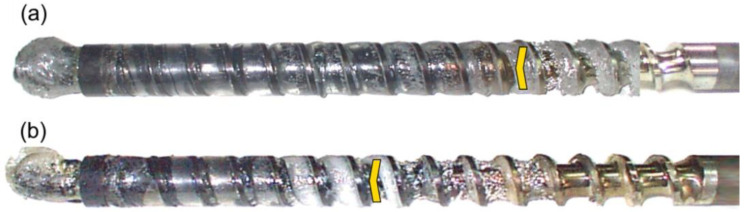
An effect of the plastication stroke on the polymer flow in the injection molding process at the screw speed N = 100 rpm, and the back pressure P_back_ = 2 MPa: (**a**) h_plast_ = 1 D, (**b**) h_plast_ = 3 D.

**Figure 13:**
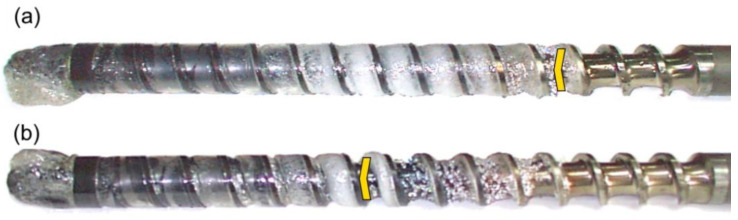
An effect of the plastication stroke on the polymer flow in the injection molding process at the screw speed N = 300 rpm, and the back pressure P_back_ = 0 MPa: (**a**) h_plast_ = 1 D, (**b**) h_plast_ = 3 D.

**Figure 14:**
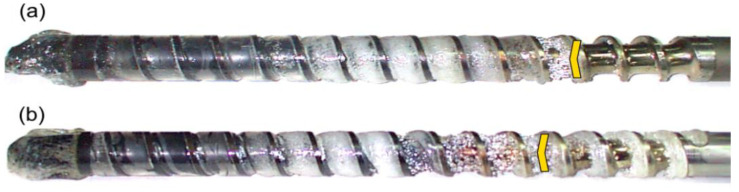
An effect of the plastication stroke on the polymer flow in the injection molding process at the screw speed N = 300 rpm, and the back pressure P_back_ = 2 MPa: (**a**) h_plast_ = 1 D, (**b**) h_plast_ = 3 D.

**Figure 15:**
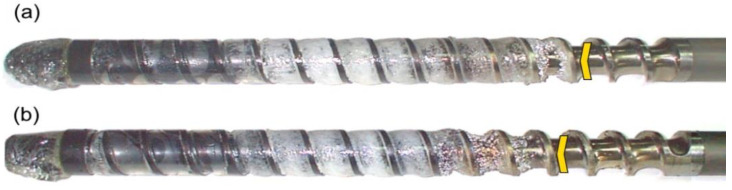
An effect of the plastication stroke on the polymer flow in the injection molding process at the screw speed N = 300 rpm, and the back pressure P_back_ = 3 MPa: (**a**) h_plast_ = 1 D, (**b**) h_plast_ = 3 D.

An effect of the plastication stroke (h_plast_ = 1 D, h_plast_ = 3 D) on the polymer flow in the injection molding process at the constant screw speed and the constant back pressure is shown in Figure 11, Figure 12, Figure 13, Figure 14 and Figure 15. It is seen that when the plastication stroke increases, the starvation increases, too.

**Figure 16:**
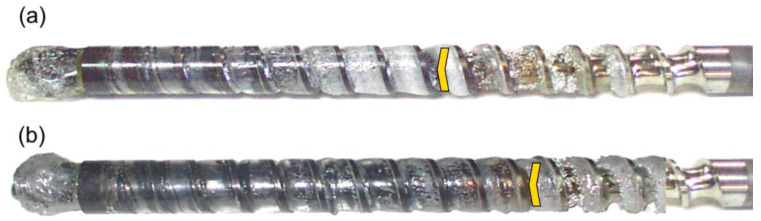
An effect of the back pressure on the polymer flow in the injection molding process at the screw speed N = 100 rpm, and the plasticating stroke h_plast_ = 1 D: (**a**) P_back_ = 0 MPa, (**b**) P_back_ = 2 MPa.

**Figure 17:**
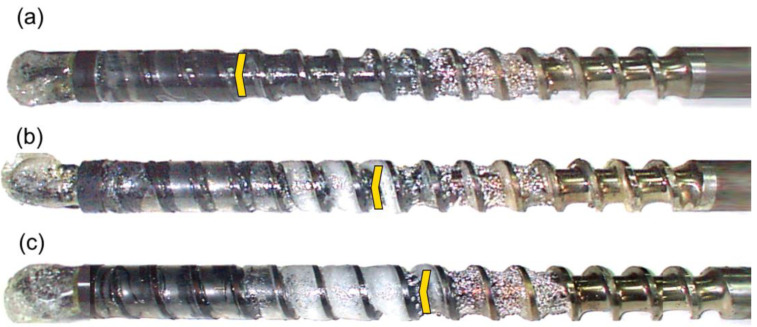
An effect of the back pressure on the polymer flow in the injection molding process at the screw speed N = 100 rpm, and the plasticating stroke h_plast_ = 3 D: (**a**) P_back_ = 0 MPa, (**b**) P_back_ = 2 MPa, (**c**) P_back_ = 3 MPa.

**Figure 18:**
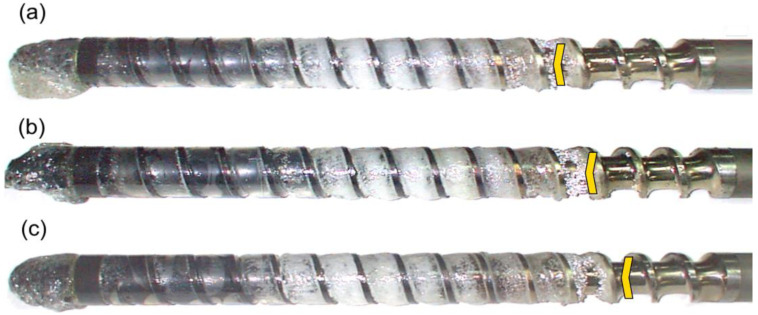
An effect of the back pressure on the polymer flow in the injection molding process at the screw speed N = 300 rpm, and the plasticating stroke h_plast_ = 1 D: (**a**) P_back_ = 0 MPa, (**b**) P_back_ = 2 MPa, (**c**) P_back_ = 3 MPa.

**Figure 19:**
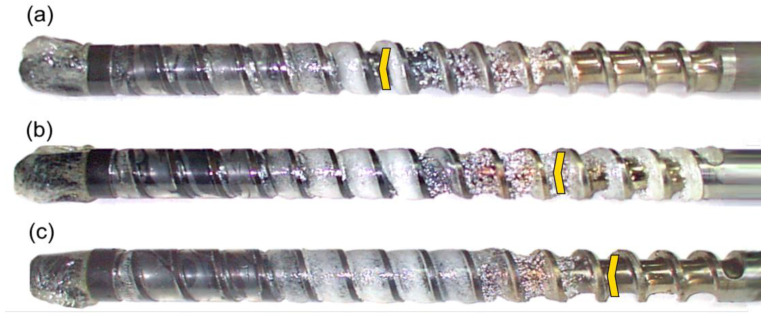
An effect of the back pressure on the polymer flow in the injection molding process at the screw speed N = 300 rpm, and the plasticating stroke h_plast_ = 3 D: (**a**) P_back_ = 0 MPa, (**b**) P_back_ = 2 MPa, (**c**) P_back_ = 3 MPa.

An effect of the back pressure (P_back_ = 0 MPa, P_back_ = 2 MPa, P_back_ = 3 MPa) on the polymer flow in the injection molding process at the constant screw speed and the constant plastication stroke is shown in Figure 16, Figure 17, Figure 18 and Figure 19. It is seen that when the back pressure increases, the starvation decreases.

It can be concluded from the presented experiment that starvation appears in the injection molding process when the screw moves forward, injecting the polymer into the mold. This is dependent on the screw speed, the plastication stroke and the back pressure. The starvation increases when the screw speed and the back pressure decrease, and the plastication stroke increases. It is also clearly seen that melting slows down when screw speed increases which is consistent with Gao’s observation [105].

## 3. Future Concepts of Injection Molding Process Modeling

There are distinct differences between the polymer flow and melting in injection molding machines and extrusion machines (reciprocating machines vs. non-reciprocating machines). In the typical cycle of injection molding, the screw starts the process in the forward position in the barrel. It rotates (screw recharge) and conveys the polymer forward, and the pressure is developed before the screw tip until the desired volume of the molded part is reached. The screw is then idled in the back position while the polymer previously injected into the mold is being cooled. After cooling the part, the mold opens, and the part is ejected. Then, the mold closes, and the screw moves forward by hydraulic pressure, injecting the newly melted portion of the polymer collected at the tip of the screw into the empty mold, and then starvation appears. The valve, such as the check ring, prevents the back flow during injection. The injection molding cycle is composed of three stages: feeding (screw rotating and moving backward), stopping (no screw movement) and injecting (screw moving forward without rotation).

The injection molding process is performed both in the plasticating unit of the injection molding machine and in the mold. To some extent, it is similar to extrusion; however, extrusion is a continuous process in which the extruder cooperates with the die continuously, without any interruptions. Injection molding is a cyclic process, and the cooperation of the injection molding machine (plasticating unit) with the mold is not continuous. However, the flow in the plasticating unit influences the flow in the mold, similarly to the extrusion, where the flow in the extruder (plasticating unit) influences the flow in the die. In the extrusion and injection molding process, the process output parameters in the plasticizing unit are the process inputs for the flow in the die and in the mold, respectively.

The conclusion is that the correct modeling of the injection molding process, and the correct modeling of the flow in the injection mold, would require including the modeling of flow in the plasticating unit into the global model of the process.

It results from the presented experiment that when injecting the polymer, the starvation appears, and this should be included in the process modeling. This can be done by adopting the concepts developed by Wilczyński et al. [8,36,44] for starve fed single screw extrusion.

In general, the models of solid conveying in the classical single screw extrusion with gravitational (flood) feeding are based on the work of Darnell and Mol [5], that is, on the assumption that the polymer granules are transported as a solid bed without relative movement of the granules. However, some experiments, for example, performed by Fang et al. [145], indicated that this cannot be valid any longer since a relative movement of the individual granules in the screw channel is clearly seen. Thus, in order to model the solid transport in the single screw extruder, the discrete element method (DEM) was applied. This was first performed by Potente and Pohl [146], who studied the polymer flow in the hopper region in the extruder. Later, important and fundamental studies were carried out by Moysey and Thompson [147,148,149], who performed the first computations for compacting granules and the pressure/throughput relation in the feed zone of the single screw extruder [150]. Further studies of this issue were performed by Schöppner et al. [151,152], who proposed the model which enabled the computation of solid conveying with considering the pressure build-up and the filling degree. It can be concluded that the discrete element method (DEM), which is a useful and powerful tool for modeling the solid conveying in single screw extruders, could also be applied to modeling this in the injection molding process. A good example of the discrete element method software is EDEM [153].

Similarly, as the model of Darnell and Mol [5] for solid conveying, the model of Tadmor and Klein [20] for polymer melting was usually the basis for modeling the extrusion process, as well as the injection molding. However, the models of melting presented so far were mainly based on the a priori assumed melting mechanism. Instead of the melting mechanism assumed, the polymer melting in single screw (non-reciprocating) extruders can be modeled by solving the conservation equations of mass, momentum and energy. This concept was presented first by Viriyayuthakorn and Kassahun [154], who proposed the 3D model without assuming any particular melting mechanism. The phase change problem was solved using the functional dependence of the specific heat on the temperature. The solution of equations of momentum and energy provided the solid/melt distribution, which was determined by the temperature distribution. Syrjala [155] carried out the 2D simulation of the polymer melting without any melting mechanism assumed. However, in both cases, the computations were not validated experimentally.

Later, Altinkaynak et al. [156] carried out the experimental and theoretical research on modeling of polymer melting using this approach. The two-phase solid/melt polymer flow was studied with the Cross-WLF rheological model, which allowed us to consider the solid material as a high-viscous fluid while the molten material as a low-viscous fluid. Hopmann et al. [157] solved the equations of momentum and energy using the finite volume method (FVM) with the Carreau rheological model. Kazmer et al. [158] used this approach to modeling the polymer melting in barrier screws, as well as Lewandowski and Wilczyński in conventional screws [159]. This approach can be probably also applied for modeling the polymer melting in the injection molding process, e.g., using the Ansys Polyflow software [160].

It is worth noting that when modeling the injection molding process, the three separated phases of the process have to be considered, i.e., the feeding phase, the idle phase and the injecting phase, and the concepts presented above should be applied separately to these.

The promising concept would be the coupled DEM/CFD modeling. Recently, EDEM and Ansys have developed an open-source, two-way coupling between EDEM and Ansys Fluent to provide a unique capability to model particle/fluid flows.

## 4. Conclusions

The issue of modeling of polymer injection molding has been reviewed and discussed, which includes both the plasticating unit and the injection mold. It has been noted that a comprehensive (global) concept of modeling of injection molding might be useful for simulating the polymer flow in injection molds, and the global model of the process might be considered for simulating the polymer flow in the plasticating unit as well as in the mold. The output parameters of the plasticating unit computations would be the input parameters for the mold flow computations.

It has also been concluded that the existing mathematical models of injection molding have no strong experimental basis. Thus, experimentation of the polymer flow and melting in the injection molding machine have been performed, and the effect of processing conditions: screw speed, plasticating stroke and back pressure on the process course has been studied. Starvation has been discovered, which was not presented in the literature so far, and which may considerably influence the process modeling. The novel concepts of injection molding modeling have been discussed. The DEM/CFD concept has been indicated as promising.

## Data Availability

The data presented in this study are available on request from the corresponding author.
